# On the Chemical Origin of Biological Cognition

**DOI:** 10.3390/life12122016

**Published:** 2022-12-03

**Authors:** Robert Pascal, Addy Pross

**Affiliations:** 1 Laboratoire de Physique des Interactions Ioniques et Moléculaires (PIIM), Aix-Marseille Université—CNRS, 13013 Marseille, France; 2 Department of Chemistry, Ben-Gurion University of the Negev, Be’er-Sheva 8410501, Israel

**Keywords:** origin of life, dynamic kinetic stability, thermodynamic stability, cognition, molecular replication, evolution, consciousness

## Abstract

One of life’s most striking characteristics is its mental dimension, one whose very existence within a material system has long been a deep scientific mystery. Given the current scientific view that life emerged from non-life, how was it possible for ‘dead’ matter to have taken on mental capabilities? In this Perspective we describe the existence of a recently discovered non-equilibrium state of matter, an energized dynamic kinetic state, and demonstrate how particular chemical systems once activated into that kinetic state could manifest rudimentary cognitive behavior. Thus, contrary to a common view that biology is not reducible to physics and chemistry, recent findings in both chemistry and biology suggest that life’s mental state is an outcome of its physical state, and therefore may be explicable in physical/chemical terms. Such understanding offers added insight into the physico-chemical process by which life was able to emerge from non-life and the perennial ‘what is life?’ question. Most remarkably, it appears that Darwin, through his deep understanding of the evolutionary process, already sensed the existence of a connection between life’s physical and mental states.

## 1. Introduction

Though the nature and origin of life have been a source of wonder for centuries, it is only in the early part of the last century, that these problems began to be addressed in a scientific manner [1,2,3]. Inspired by Darwin’s theory of evolution [4], significant advances followed in rapid succession. The merging of Darwin’s theory of natural selection with Mendel’s ideas on heredity into what is often termed neo-Darwinism could be considered the beginning of a process of biology’s physicalization. That physical approach was subsequently reinforced by Schrödinger’s classic “What is life?” text [5], which clarified how far-from-equilibrium chemical systems could be maintained over time through the continual utilization of energy. Watson and Crick’s 1953 landmark discovery of DNA’s molecular structure [6] and Stanley Miller’s exploration of prebiotic chemistry [7] followed soon after. Increasingly, biology appeared to be amenable to physical reduction and characterization. More recently, Ganti [8] built on that reductionist approach by attempting to characterize minimal life through the identification of its three sub-systems—a self-replicating informational system, a metabolic system able to provide chemical building blocks and energy, and a physical compartment that encloses the cell’s component parts. Thus, having been triggered by Darwin’s landmark theory of evolution, the physicalization of biology seemed to be well underway. Life and its emergence were increasingly understood to be the consequence of objective physical law [9,10].

However, deep concerns regarding the physicalization process have continued to overshadow the field. As pointed out by several of the leading physicists of the last century, life somehow seemed to transcend its material constraints, and several were led to question the reductionist thesis [5,11,12]. In fact, building on that anti-reductionist view, the noted philosopher Thomas Nagel recently went as far as to question reductive materialism as a legitimate philosophic basis for scientific understanding [13]. Awkwardly, despite the enormous biological advances since Darwin, the life phenomenon continues to be physically obscure, the physics–biology relationship remaining as contentious as ever.

At the heart of the problem lies the issue of agency, life’s undeniable purposeful character. Leading 20th-century biologists, such as Monod [14] and Dobzhansky [15], stated the difficulty explicitly. Organisms do not act as passive material forms responding to the dictates of natural law but act in a purposeful, goal-directed manner. As Dobzhansky pointed out: “Purposefulness, or teleology, does not exist in nonliving nature. It is universal in the living world. It would make no sense to talk of the purposiveness or adaptation of stars, mountains, or the laws of physics.” However, how can passive material forms, solely governed by objective physical law, act on their own behalf? At what point in the evolutionary process from inanimate toward life was that teleological Rubicon crossed? Was the transformation gradual or was there a discrete phase transition at some point? Attempts to marginalize such purposeful behavior as “seemingly goal-directed” [16] or as “powerful illusions” [17] were expressed, but more recent studies [18,19,20], in particular on bacterial life [21,22,23,24], have indicated that such explicit purposeful behavior is central to *all* biological activity. Therefore, what then is the physical basis for such unambiguous purposeful, goal-directed behavior?

Central to life’s operational goal-directed capabilities lies that of cognition. Indeed, Maturana and Varela hypothesized that cognition was at the heart of all biological activity and that “living as a process is a process of cognition” [25]. However, the same physical question arises yet again: how can matter of any kind be cognitive? If, indeed, life did emerge from non-life, as is now generally accepted, how was ‘dead’ matter able to become cognitive? What laws of nature could explain the transformation of matter from being inanimate and passive to becoming cognitive and active? Could the physical sciences be in some sense incomplete [26]?

In this article, we will describe new developments in both chemical theory and practice that point to the existence of a new dimension in chemical potentiality, one whose existence only became more evident a little over a decade ago. It is through the discovery of this new material dimension that the origin of life’s striking biological characteristics—agency, cognition, function—is now coming to light. That development can be considered part of a newly emergent area in chemistry, systems chemistry [10,27], one which deals with *networks* of reacting molecules rather than with the reactions of individual molecules. We will attempt to demonstrate that despite long-standing reservations, life’s unique and contentious non-material characteristics may have an identifiable physical basis.

## 2. Discussion

### 2.1. Emergent Properties of Matter

Let us begin our analysis with the reaffirmation that emergent properties can often (though not always) be understood through reductionist thinking. That, broadly speaking, has been the basis of the scientific method for centuries [28] and it has been highly effective in its ability to explain matter’s physical and chemical characteristics. Thus, the fact that water can exist as a solid, a liquid, or a gas; that metals can conduct electricity while plastics cannot; that table salt dissolves in water but not in oil, are all emergent material properties that are readily explained in reductionist terms. In fact, it was reductionist thinking that led to the uncovering of two of nature’s key physical laws—the late 19th-century formulation of the Second Law of Thermodynamics and its underlying kinetic theory of matter. Crucial to that formulation was the growing realization in the latter part of the 19th century that molecular entities exist. Without the hypothesis of molecularity, the conceptual leap associated with those two central laws could not have been taken. We emphasize this point to make it clear that successful reductionism invariably depends on certain prior knowledge, and without such prior knowledge, the methodology cannot operate.

However, here, we run into a paradox. Given the importance of the concept of molecularity for our understanding of material behavior, it would be natural to expect that molecular reductionism would also be able to lead to an understanding of the life phenomenon. After all, are not all living things just complex molecular aggregates? Yet, somehow, a molecular approach to life has not been able to resolve the life enigma. Though the spectacular advances in molecular biology of past decades have unambiguously reaffirmed life’s molecular underpinnings, those dramatic advances have only served to increase the conceptual mystery. As Kauffman put it: “… we know many of the parts and many of the processes. But what makes a cell alive is still not clear to us” [29]. Life’s central question—what is it?—continues to torment. Life’s unique emergent properties have remained resistant to attempts to reduce them to physics and chemistry. Indeed, as noted earlier, a prevailing view amongst both physical and biological scientists is that biology is *not* reducible to physics and chemistry [5,11,12,30,31].

So where does the problem lie? Is material reductionism a flawed conceptual methodology, at least as it pertains to biology [13]? We do not believe so, though reductionism, like all methodologies, obviously has its limitations. Clearly, for higher-order emergent phenomena to be explained in lower-level terms, the appropriate lower level of organization needs to be considered. One cannot, for example, hope to explain social behavior in molecular terms or material phenomena in sub-atomic particle terms. The hierarchical nature of scientific explanation and the inherent limits of reduction need to be taken into account [32].

Therefore, if applied appropriately, could biological phenomena be reducible to physics and chemistry? We believe the answer is *yes*, though the devil lies in the term ‘appropriately’. Unexpectedly, in a life context, reduction to the molecular level may not be the optimal level, at least for understanding life’s striking emergent properties. Molecularity is, without doubt, the appropriate reductionist level for explaining many of biology’s cellular processes—ribosomal protein synthesis, ATP synthesis, DNA replication, and many more, but it turns out that matter’s molecular building blocks are not the appropriate ones for understanding life’s *global* characteristics. As we will now discuss, for an understanding of life’s global characteristics, a higher level of molecular organization is required, one based on the existence of *energy-fueled, dynamic molecular assemblies*, chemical systems with the ability to act autonomously [33,34]. Indeed, it was the recent discovery that such energized dynamic molecular assemblies have a discrete physical existence that led to the uncovering of a new dimension in chemical possibility—the domain of what was termed dynamic kinetic chemistry [35,36]. It is within that new but unfamiliar chemical domain that living things belong, thereby opening up new insights into the physical basis for the life phenomenon together with its striking emergent properties.

### 2.2. The Discovery of Dynamic Kinetic Chemistry and the DKS State

The nature of change in the physical world has been well understood since the late 19th-century formulation of the ubiquitous Second Law of Thermodynamics [37]. Thus, for any chemical process in which two substances X and Y interconvert, the relative proportions of X and Y in the reaction mixture will undergo no further change (under specified conditions), once a state of equilibrium, designated as X⇄Y, is achieved.

However, nature is replete with non-equilibrium systems, and landmark work by Prigogine and co-workers on the nature of such systems enabled thermodynamic understanding to be broadly extended to such systems as well. Thus, the concept of “dissipative structures” was proposed—ordered and stable non-equilibrium systems that can be maintained through energy and material transfer with their environment [38]. The relevance of Prigogine’s ideas to the life phenomenon became immediately clear as all living things exist in a non-equilibrium state. Accordingly, it was proposed that living things could be usefully thought of as “dissipative structures”, and, consistent with that underlying thermodynamic perspective, life was but an additional mechanistic pathway by which nature was able to dissipate energy [39]. Yet, despite that added thermodynamic insight, the physical basis for life’s emergent properties remained no less mysterious.

**Figure 1:**
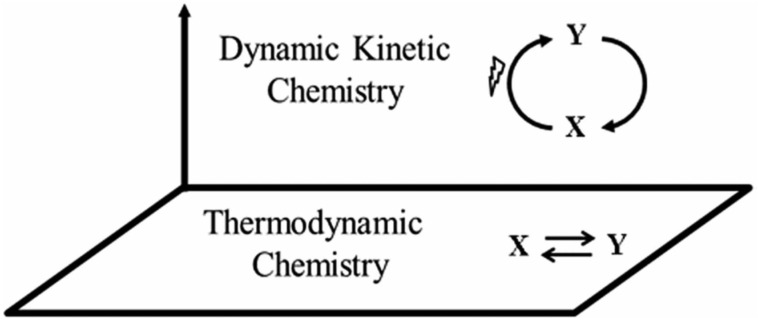
Schematic display of the two general domains in chemistry, the established thermodynamic domain, and the recently characterized dynamic, energy-fueled, kinetic domain [35,36]. Note that for any single thermodynamic system X⇄Y, kinetic factors allow for the in principle existence of an infinite number of energized kinetically governed systems, which together constitute the corresponding kinetic domain.

It has become increasingly apparent in recent years that kinetic factors also play a crucial role in governing life processes. The roots of that kinetic view can be traced back to the work of Lotka [40] who, in attempting to understand the physics of life, pointed out that the laws of thermodynamics “*tell us that certain things cannot happen, but they do not tell us what does happen*”. In traditional physical thinking, systems are termed kinetically stable when the system is located within a local energy minimum, one separated from the system’s lowest energy state by an energy barrier. For such systems, the kinetic stability is of a static kind and that kind is well understood in chemical theory. However, there exists an alternative kind of kinetic stability, a *dynamic* one, that was termed *dynamic kinetic stability (DKS)* [41,42,43,44,45], and, as will now be discussed, that less familiar kind of stability was found to bear directly on both chemical and biological reactivity patterns [46,47,48,49,50,51]. Indeed, it is within that kinetic domain that novel autonomous chemical systems can be found. The relationship between kinetic space and thermodynamic space—two coherent dimensions of chemical possibility—is illustrated schematically in Figure 1. As indicated in the figure, the interconversion of X and Y in a time-stable energy-fueled dynamic state can take place independently of one based on their intrinsic thermodynamic relationship. Accordingly, *two* distinct chemical worlds exist, one that could be termed ‘regular’ thermodynamic chemistry, and an alternative one of dynamic kinetic chemistry [35,36]. Crucially, the kinetic domain is more extensive (vastly so) than the thermodynamic one, and living things—stable energy-fueled, non-equilibrium structures—can now be understood as material forms that occupy that energized kinetic domain [43,44,45,46,47,48,49,50].

Importantly, however, for the DKS state to be maintained, a continuing source of energy needs to be supplied and that energy supply is a central element of the DKS state description. To help understand the DKS concept, it is useful to think of a simple physical DKS system—a water fountain—as it expresses several of the key characteristics of the chemical DKS state—it is dynamic, energy-fueled, non-equilibrial, yet stable. The water fountain can be maintained indefinitely provided water is continually available and the pump that operates the fountain is continuously supplied with energy [42,43,44].

The landmark chemical experiment that underscored the existence of *chemical* DKS systems was reported a decade ago by van Esch, Eelkema, and colleagues [52,53]. Their study was based on a modification of one of organic chemistry’s most conventional reactions, esterification. Whereas esterification is normally carried out as a thermodynamically directed process, a different material outcome resulted when a continual input of matter and energy was directed into the reaction system. A kinetically stable hydrogel product was obtained, one quite different in its properties to the conventional thermodynamic product. Significantly, that experiment made clear that there exist *dynamic kinetic states of matter*—dynamic, energized, thermodynamically unstable, yet persistent material forms [47]. In some sense, their discovery could be thought of as extending Prigogine’s physical “dissipative structures” into the chemical arena. Thus ‘chemical fountains’ exist—chemical systems in an energized dynamic kinetic state—and their physical basis was characterized in some detail [54,55,56,57]. In recent years, many examples of such systems have been reported, opening up new synthetic possibilities in material science [58].

However, in the context of life processes, the existence of chemical DKS systems is of paramount importance as they provide direct chemical support for Maturana and Varela’s theory of autopoiesis [25]. That theory characterizes life as a dynamic cyclic process that maintains itself by creating its own components. What we learn from the DKS formulation is that the general autopoietic formulation can be expressed in quantifiable kinetic and energy terms. Thus, autopoietic living systems can be thought of in physicochemical terms as dynamic energized kinetically stable chemical systems [54,55,56,57], thereby strengthening the biological perspective. Moreover, such physicochemical characterization can help explain life’s unique characteristics several of which remain mysterious through the biological perspective alone. Thus, it is easier to understand why such energized systems are readily deactivated, a process that in the life context we term death, while the reverse process, to re-activate the system back into that energized state is far more elusive, a continuing chemical challenge. A merging of the insights from DKS chemical thinking together with those from Maturana and Valera’s theory of autopoiesis leads to a more comprehensive understanding of the life phenomenon.

However, now to the central life question at the heart of this paper: how can the existence of autonomous dynamic kinetically stable systems be helpful in our attempts to understand life’s striking emergent properties, in particular, that of cognition? The DKS state on its own is insufficient to fully understand the nature of the living state. An added requirement is that the system also possesses a replicative capability as only then could a Darwinian-like evolutionary process take place. Indeed, since the landmark work of Spiegelman in the 1960s [59], it is now well established that certain molecular systems are able to undergo a replication reaction, whether through a template-type mechanism [60] or through the establishment of an autocatalytic cycle [29]. However, it is only when such a system is activated into the DKS state that an evolutionary process from *less* DKS stable to *more* DKS stable can take place [47,60,61], thereby identifying the directive underpinning the evolutionary process. Thus, contrary to conventional biological thinking [62], there is a general direction for evolution, one specified by the system’s kinetic stability. Observe also that the basis for biology’s central principle of natural selection can be found in the kinetic selection behavior of DKS systems; natural selection in effect is just the biological term for kinetic selection [43,47].

Let us now return to the issue of life’s cognitive character and explore how its emergence could become explicable in physico-chemical terms.

### 2.3. Cognition’s Physical Basis

Though there is no widely accepted definition for cognition [63,64,65,66], a useful definition proposed by Shettleworth is that cognition is “the mechanisms by which living things acquire, process, store, and act on information from the environment” [67]. That definition, initially proposed for neural systems, was unexpectedly later found to be equally applicable to aneural life, even the simplest bacterial life. In support of that revised view, recent studies by Shapiro [21,22] and Lyon [23,68] have described how pervasive and sophisticated cognitive behavior in bacteria can be. This simplest of life forms is able to sense its internal condition, coordinate with neighboring organisms, and routinely activate elaborate response systems in response to ongoing challenges. Thus, remarkably, life’s mental capability is already apparent at that simplest life level. As Shapiro put it: “bacteria are small, but not stupid” [22].

An intriguing observation regarding life’s early mental capabilities is that Darwin already alluded to such capabilities. As pointed out by Lyon, Darwin hypothesized that life’s mental capabilities were there from life’s beginnings and that the evolutionary process from its outset proceeded along both physical *and* mental axes [69]. However, if indeed life’s mental character is inherent in all life, right down to the simplest life forms, this has clear implications with regard to the origins of that mental character. It would suggest that the process of mental evolution did not begin with the earliest life but would have existed in some earlier prebiotic *chemical* system. In other words, cognition would have been initiated in chemistry.

At first sight, such a statement might sound quite unreasonable. How could *any* chemical system express cognitive character, though to be clear we are not arguing for the existence of active mental activity at that early chemical stage. The term ‘mind-like’, coined by Varela, would be more appropriate [70]. However, if cognition was associated with the simplest life, as is now widely believed, evolutionary logic strongly suggests that cognition, at least in rudimentary form, would have been initiated in chemical pre-life. After all, the simplest life did not just appear out of the blue. It would have presumably evolved from an earlier evolutionary stage, one we would classify as being chemical. Continuity, after all, is at the very heart of Darwinian thinking [42].

However, one might then ask, if cognition did begin in chemistry, what kind of chemical system might express rudimentary cognitive function? How was the cognitive Rubicon, from inanimate passive to cognitive active, crossed? In the following section, we will describe how the replicative DKS state, being common to both chemical and biological systems, might throw light on the physical basis of cognition and the possible origin of life’s mental state.

### 2.4. Cognition and the Replicative DKS State

All material systems, whether living or non-living, respond to external perturbation, though each kind does so in distinctly different ways. Thus, non-living matter responds to perturbations through the directing effects of the Second Law. The heating of a physical object might cause it to expand or undergo a phase transition, while perturbing a chemical system at equilibrium, by adding an additional reagent or changing the concentration of one of its constituents, would cause it to seek to re-establish that equilibrium state. In both physical and chemical cases, the system’s response to the perturbation would be explicable in thermodynamic terms.

Living systems behave very differently. Though they of course fully obey the physical laws of nature, in practice they follow biological principles, and, accordingly, respond to perturbations through what is termed adaptation; organisms respond to perturbations in a way that supports the organism’s agenda. Consider chemotaxis, for example, the directed motion of an organism in response to some stimulus [71]. Such motion is understood as resulting from the organism’s agenda of survival rather than being governed by physical/chemical principles. Accordingly, a bacterium ‘swims’ toward nutrition but away from toxins. A biological system’s response to perturbations, though of course fully consistent with the Second Law, is not *explained* by the Second Law. Adaption is necessarily in accord with thermodynamic constraints but cannot be predicted through thermodynamic considerations. However, living things are ultimately physical/chemical systems, therefore the question remains: could life’s adaptive nature be explicable in more fundamental physical/chemical terms, rather than in the more usual biological ones? As we will now discuss, the DKS concept may offer fresh insights into this fundamental question.

In contrast to inanimate systems, living systems are crucially dependent for their sustainability, in fact, their very existence, on their environment. Photosynthetic life depends critically on a continual supply of solar energy, carbon dioxide and water, while aerobic life depends critically on a continual supply of oxygen and chemical resources. For living systems, the material and energy resources on which they depend are not simply perturbations, they are crucial support systems that enable those living things to stay alive. This description of biological dependence is consistent with the DKS description of a chemical system. Thus, all DKS systems, whether physical or biological, are in that same way totally dependent on a continuing supply of material and energy resources. To help clarify this point, let us return to the physical example of a water fountain. As discussed earlier, the fountain’s stability, its very existence, in fact, depends on the continual supply of water and energy to the pump that maintains the fountain. Interrupt either and the fountain ceases to exist. In that sense, the impact of those resources is quite distinct from perturbations in the thermodynamic sense.

Clearly then, the relationship between a DKS system and its environment is quite different from that of an inanimate system to its environment. The DKS system and its supportive environment are intimately connected, with the DKS system’s very existence being dependent on the continuity of that connection. Indeed, that umbilical connection between the system and its supportive environment creates what is effectively an ‘inside’ linked to its ‘outside’. Observe that the ‘inside-outside’ relationship between the DKS system and its environment emerges without the need for a compartment or separation barrier. The relationship is *ontological* rather than *structural*. In fact, the usual structural manifestation of that ontological relationship—the cell membrane—can now be understood as coming about through an evolutionary process that leads to the functional requirements that arise from that ontological relationship. In other words, the physical emergence of the cell membrane in evolution could be understood as a material *consequence* of that ‘inside-outside’ ontological relationship rather than its *cause*. Observe also that through the ‘inside-outside’ description of a DKS system connected to its environment, the concepts of ‘self’ and ‘non-self’ in a life context appear naturally. Thus the biological concepts of autopoiesis and cognition, traditionally viewed from a biological perspective [72], are now addressed in physical/chemical terms.

Continuing with this DKS perspective, let us now consider how life’s mental dimension could have emerged from a physical system. The fact that a physical system (the DKS system) is *existentially dependent* on another physical system (its supportive environment) can be viewed as the beginning of a mental state. *Dependence* is a non-material state and it can be thought of as expressing a rudimentary mental state, and following a similar line of reasoning, the origin of cognition, which is defined as a mental phenomenon, can be seen to derive from the dynamic and continuing exchange of material and energy between the DKS system and its supportive environment. In other words, a physical/chemical mechanism for the emergence of mental (non-material) phenomena—*dependence* and *cognition*—emerges from the physical nature of the DKS state. Furthermore, through the ‘inside-outside’ interdependence, the beginnings of information transfer in the biological sense can be discerned. The continual flow of essential resources from the environment to the DKS system, and its response to that flow, can be interpreted in informational terms.

However, let us now return to the crucial role of replication in life and examine how it relates to the preceding discussion. Once a DKS system takes on a replicative capability, kinetic analysis of such a system indicates that an evolutionary process is able to ensue [60,61]. However, as will now be discussed, it is that replicative process that eventually leads to the emergence of explicit cognitive character and the beginnings of agency. To be clear, it would not be appropriate to associate a *non-replicative* DKS system with a rudimentary sense of self or cognition. A physical DKS system, such as a water fountain or a chemical DKS system, such as van Esch’s DKS ester system, would not be reasonably classified as cognitive, despite them being intimately linked to their environment. It would only be through an evolutionary process, whereby a replicative system would be able to evolve, that explicit cognitive function could begin to emerge—*perception*, *memory*, and *learned response*. Perception would come to be through the dynamic interplay between the DKS system and its supportive environment. Memory is inherent within the replicative process, as it is through such a process that favorable heritable characteristics over successive generations can be maintained. The kinetically directed process by which less stable DKS replicators are continually replaced by more stable ones could be understood as a manifestation of ‘learned response’. Through the process of kinetic selection, the system ‘learns’ which variational forms are advantageous, and which are not. Thus, a DKS system that is replicative has the capacity to both ‘remember’ and ‘learn’, clear expressions of a mental dimension. Accordingly, the DKS concept enables the chemical roots of life’s mental aspects to be discerned and understood in physico-chemical terms. Moreover, just as Darwin intimated in his Origins, the evolutionary process would appear, from its outset, to have encompassed life’s mental facet and not just its physical one. Life’s mental facet can now be understood as being an integral aspect of the replicative DKS state.

Observe also that such an evolutionary process would also lead to the DKS system undertaking material modification of its environment through what biologists have termed ‘niche construction’ [73]. In effect, the ‘inside’ undertakes modifications of its ‘outside’ through the same process of kinetic selection that enables internal modification. All kinetic selection in DK space is necessarily directed toward one goal—enhanced DK stability, just as all modifications in regular physical/chemical space are directed toward enhanced thermodynamic stability.

From the above discussion, the distinction between the organization of a living organism and a mechanical entity such as a watch becomes clearer. A DKS system is not a mechanical, machine-like entity such as a watch. It is a dynamic energized system with an obligatory dependence on its surroundings with inherent cognitive capabilities. Thus, we would argue that the emergence of a replicative chemical DKS system, able to evolve in an open-ended manner, would have been the watershed moment that led to the emergence of a process leading to material life together with the non-material accompaniment of cognition. That view is fully consistent with Maturana’s insightful comment described earlier, that “living as a process is a process of cognition” [25] and supports the idea that life’s physical *and* mental facets are intimately linked and materially based.

As a final point, it should be emphasized that the DKS term characterizes a *population* of entities, not individual ones, whose existence is necessarily transient and part of the dynamic process on which the DKS system is based. Indeed, in that population context, the role of death becomes clearer. Death is not just something unfortunate that happens to living things but is a central feature of the DKS cycle. Without death, there can be no life. Death facilitates the dynamic process of population renewal, enabling the continual scouring for new variants able to offer improved function, thereby leading to increased DKS. Ultimately DKS enhancement, by whatever means, is the evolutionary name of the game.

To sum up, though cognition has intrinsically non-material aspects, it derives from material causes and is able to induce material consequences in both the system and its environment. Indeed, that is why cognition emerged. From this perspective, neither cognition nor its associated mental counterparts—agency, self, mind—can exist independently of their material base. Nature ‘discovered’ and ‘learnt’ to exploit the mental dimension in the same way that it discovered and learned to exploit the physical dimension—to facilitate nature’s ultimate drive, the drive toward increasingly persistent forms [47,48]. Biology’s fundamental chemical building block can now be specified—material systems in the DKS state, a non-equilibrium energized state, and it is at that level of chemical organization that life’s physical and mental capabilities can finally emerge from the chemical shadows. The actual physical pathway by which early mental manifestation was able to evolve into the full-blown consciousness of advanced life forms is likely to preoccupy researchers for decades to come.

## 3. Concluding Remarks

Darwin’s theory of evolution, based on the idea of natural selection, was a landmark event in the natural sciences for the unity it brought to biology—the profound insight that all living things were related to one another. However, no less significantly, Darwin’s theory was the first step in an attempt to bridge between the physical and biological worlds, or, expressed more pointedly, to physicalize biology. However, a century and a half on, that process of physicalization seems to have stalled. Paradoxically, the Darwinian revolution, alongside its landmark revelations, exposed deep contradictions within the natural order, and to the present time, these contradictions remain unresolved. Life’s unique characteristics—function, agency, purpose, mind—remain inconsistent with the traditional physical view of an objective universe governed by natural law. Biology, the autonomous science [16], continues to remain troublingly conceptually separated from the physical sciences.

However, as described earlier, physicalization effectively rests on applying a reductionist methodology, and the methodology can only work if it is carried out appropriately. What is now increasingly clear is that the right level of reduction for understanding certain aspects of the life phenomenon is not at the simpler molecular level but at a more complex level, that of energized, dynamically stable molecular aggregates. Beyond describing the physical nature of the evolutionary process—from less DKS stable to more DKS stable—that higher level of complexity appears able to explain how an evolutionary process may have been initiated from chemical beginnings. However, in the context of this article, its relevance lies in its ability to offer insights into the physical basis for life’s puzzling mental characteristics. Just as matter can express a wide range of characteristics—to be hard, soft, conductive, reflective, invisible, and so on—matter in a particular energized state can begin to manifest what could be thought of as mental characteristics. We attempted to demonstrate that replicative systems in the DKS state, through ongoing dynamic interaction with its environment, are able to display rudimentary signs of cognition, thereby offering insight into the likely physical origin of matter’s mental dimension. Thus, despite the common view that biology is not reducible to physics and chemistry, our analysis suggests it may well be. Biology’s successful reduction to physics and chemistry, as for all methodological reduction, requires that an appropriate level of the reduction be chosen. More generally, we would argue that reductionism within the natural sciences, both physical and biological, continues to be the key conceptual methodology for achieving scientific understanding.

Interestingly, despite biology’s apparent discomfort with reductionist thinking in some quarters, it was supported by Eric Kandel when seeking a greater understanding of what must be the most challenging biological puzzle of all, the workings of the brain [74]. Kandel observed that the neurobiological underpinnings of learning and memory in primitive organisms, such as sea slugs, were able to shine a light on the nature of mental processes in higher animals. Thus, the nature of the evolutionary process at any level, both simple and complex, appears to open itself to reductionist thinking. In fact, given the continuity from chemical through to advanced biological, one could argue that a reductionist approach might lead to insights into the ultimate biological mystery, that of consciousness [75]. The challenge, as always, is to choose the right hierarchical level of complexity for explaining the phenomenon of interest. Indeed, one might go as far as to say that to categorically deny reductionist methodology in biology is, in effect, to deny biology’s material beginnings. The fact that a physical/chemical system was able to evolve into a biological one, in and of itself, indicates that biology *is* inherently reducible to physics and chemistry.

Remarkably, Darwin in his genius already sensed the profound role of reduction in his attempt to understand life. He intuitively understood that life most likely emerged from non-life in an evolutionary process—reductionistic thinking per se. However, Darwin went further. Through reductionist thinking, he intuitively understood that the evolutionary process involved both “corporeal and mental endowments” [4], namely, that it took place along both physical *and* mental axes, that both facets would necessarily have been present from the beginning of the evolutionary process. He sensed that life’s mental aspect was unlikely to have just popped out willy-nilly from within a physical evolutionary process. The underlying logic of evolutionary continuity would argue against that. And Darwin achieved all this with only 19th-century science and his intuition to build upon. Accordingly, Lyon’s recent summary statement on Darwin’s thinking seems apt [69]: “it is time to embrace Darwin’s radical idea that our minds, too, are evolved from much simpler minds. Body and mind evolved together and will continue to do so”. It is instructive that the recent chemical developments described in this article, both experimental and theoretical, offer support for this view. The mind increasingly appears to be a particular manifestation of certain energized material forms. The conceptual path leading from physical to mental may finally be opening up.
